# Supplementary material for: Mechanisms governing the pioneering and redistribution capabilities of the non-classical pioneer PU.1
Source: Nat Commun. 2020 Jan 21;11:402. doi: 10.1038/s41467-019-13960-2 (PMC6972792; doi:10.1038/s41467-019-13960-2)
Supplement: Supplementary file 7 — Source data [file 41467_2019_13960_MOESM7_ESM.zip › Source_Data/FigureS3/FigureS3J_MotifScanOutput/homerResults/motif25.similar.html]

motif25

## Information for motif25

A
C
G
T
C
A
T
G
G
C
T
A
A
T
C
G
T
A
C
G
A
C
G
T
G
T
A
C
C
G
T
A
G
A
T
C
G
T
A
C
  
Reverse Opposite:  

C
A
T
G
C
T
A
G
A
C
G
T
A
C
T
G
G
T
C
A
A
T
G
C
T
A
G
C
C
G
A
T
G
T
A
C
G
T
C
A
  

|  |  |
| --- | --- |
| p-value: | 1e-44 |
| log p-value: | -1.034e+02 |
| Information Content per bp: | 1.636 |
| Number of Target Sequences with motif | 2529.0 |
| Percentage of Target Sequences with motif | 19.16% |
| Number of Background Sequences with motif | 5072.6 |
| Percentage of Background Sequences with motif | 13.90% |
| Average Position of motif in Targets | 421.1 +/- 388.7bp |
| Average Position of motif in Background | 316.2 +/- 204.4bp |
| Strand Bias (log2 ratio + to - strand density) | 0.0 |
| Multiplicity (# of sites on avg that occur together) | 1.15 |
| Motif File: | file (matrix) reverse opposite |

### Similar de novo motifs found

|  |  |  |  |  |  |  |  |
| --- | --- | --- | --- | --- | --- | --- | --- |
| Rank | Match Score | Redundant Motif | P-value | log P-value | % of Targets | % of Background | Motif file |
| 1 | 0.771 | G C A T A T C G T C G A A G T C T G A C G C A T T G C A C G T A A G T C A G T C G T A C | 1e-33 | -77.470883 | 6.49% | 3.82% | motif file (matrix) |
